# *Per os *administered refined olive oil and marine PUFA-rich oils reach the cornea: possible role on oxidative stress through caveolin-1 modulation

**DOI:** 10.1186/1743-7075-6-48

**Published:** 2009-11-23

**Authors:** Mélody Dutot, Hong Liang, Chantal Martin, Delphine Rousseau, Alain Grynberg, Jean-Michel Warnet, Patrice Rat

**Affiliations:** 1 Laboratoire de Toxicologie, Faculté de Pharmacie, Université Paris Descartes, Paris, France; 2 Institut de la Vision, Paris, France; 3 Lipides Membranaires et Fonctions Cardiovasculaires, Institut National de la Recherche Agronomique-UR1154, Faculté de Pharmacie, Université Paris-Sud, Châtenay-Malabry, France

## Abstract

**Background:**

Olive oil and fish oils are known to possess beneficial properties for human health. We investigated whether different oils and fatty acids alone were able to decrease oxidative stress induced on corneal cells.

**Methods:**

In our *in vivo *study, rats were fed with marine oils rich in polyunsaturated fatty acids (PUFA) or refined olive oil during 28 days. At the end of the protocol, corneas were analysed for their fatty acids composition to study the incorporation of fatty acids in cell membranes. In our *in vitro *study, a human corneal cell line was incubated with marine oils or refined olive oil and subjected to oxidative stress (tBHP 50 μM, 1 hour). Effects on reactive oxygen species generation, mitochondria and caveolin-1 expression were studied using microcytofluorometry, flow cytometry and confocal microscopy.

**Results:**

Our results indicate that dietary oils changed the fatty acids composition of corneal cell membranes. According to our results, PUFA-rich oils and refined olive oil (free of antioxidants) blocked reactive oxygen species production. Oleic acid, the major fatty acid of olive oil, also decreased oxidative stress. Moreover, oleic acid modified caveolin-1 expression. Antioxidant properties of oleic acid could be due to disruption of membrane microdomains such as caveolae.

**Conclusion:**

Oleic acid, a potential potent modulator of oxidative stress, could be added to PUFA-rich oils to prevent oxidative stress-linked corneal pathology.

## Background

Olive oil constitutes a major component of the "Mediterranean diet", referring to countries that surround the Mediterranean Sea and tend to have a low incidence of chronic degenerative disease, particularly coronary heart disease and cancers of the breast, skin, and colon [1-3]. The chief active components of olive oil include oleic acid, phenolic constituents and squalene. The main phenolics include hydroxytyrosol, tyrosol and oleuropein, which occur in highest levels in virgin olive oil and have demonstrated antioxidant activity. Antioxidants are believed to be responsible for a number of olive oil biological activities. Oleic acid, an omega-9 monounsaturated fatty acid (MUFA), has shown activity in cancer prevention, while squalene has also been identified as having anticancer effects. Besides Mediterranean diet, fish diet is also known to have beneficial effects on human health. Two long-chain omega-3 polyunsaturated acids (PUFA), the docosahexaenoic (DHA) and eicosapentaenoic (EPA) acids, are found in fatty fish and other marine sources and might be the putative dietary components thought to modify the cardiovascular risk in subjects consuming high amounts of such food [4]. After ingestion, omega-3 PUFA are distributed to every cell in the body where they are involved in a myriad of physiological processes, including regulation of cardiovascular, immune, hormonal, metabolic, neuronal and visual functions [4]. In the retina, DHA has a functional benefit, as photoreceptors rich in omega-3 PUFA show improved performance in humans [5,6]. In the cornea, very little is known about the effects of PUFA and MUFA; and yet, the ocular surface epithelial cell layers, consisting of the conjunctiva and cornea, are the initial areas protecting the eye from external agents (atmospheric oxygen and sunlight, known as causative factors of oxidative stress in biological systems). At the cell level, effects of PUFA and MUFA are mediated by changes in membrane phospholipids structure, interference with eicosanoid intracellular signalling and regulation of gene expression. Most vertebrate cells display a considerable microheterogeneity in their plasma membranes, often termed microdomain structure. Some of these microdomains are enriched in glycosphingolipids and cholesterol and are resistant to solubilization with non-ionic detergents; they are therefore called detergent-insoluble-glycolipid enriched membrane or glycosphingolipid enriched membrane. These domains, also called lipid raft, are transient molecular associations between lipid and protein components of the plasma membrane, providing a dynamic patchiness and local order in the fluid mosaic membrane [7]. Most of lipid raft-associated proteins are involved in signalling pathways. Like lipid rafts, caveolae are microdomains rich in free cholesterol and sphingolipids, and are involved in transcytosis, ptocytosis, cell signalling and cholesterol regulation [8-10]. These functions are believed to require caveolin-1, the major protein component of caveolae. Caveolin-1 interacts with signalling proteins, including G-proteins, protein tyrosine kinases and nitric oxide synthase [11-13]. Reactive oxygen species are normally generated in low amounts during respiration, the process by which molecular oxygen is reduced in the mitochondrial respiratory chain to produce ATP. Reactive oxygen species can potentially cause damage to nucleic acids, proteins and lipids; cells are then equipped with antioxidants: enzymes such as catalase or superoxide dismutase and non-enzymatic systems such as glutathione. A lack of antioxidant systems or an overproduction of radicals can lead to an unbalance between oxidants and antioxidants. In this case, oxidative stress generates cell death through apoptosis or necrosis [14,15]. Gniadecki et al. wrote that the plasma membrane takes part in the regulation of oxidative stress [16]. Therefore, reorganization of the plasma membrane through caveolae remodelling could have consequences on oxidative stress. Our hypothesis is that exogenous fatty acids can incorporate into cell membranes, remodel lipid raft domains and influence the expression of proteins contained in these domains such as caveolin-1, leading to a possible antioxidant effect.

The aim of this work was first to study the effect of *per os *administration of different oils on the cornea, and second to compare and understand their ability to decrease oxidative stress induced on cultured corneal epithelial cells.

## Methods

### Reagents

PUFA-rich oils (microalgae DHA, fish EPAX 1050 and fish EPAX 6000) were purchased from Yslab (Quimper, France) and refined olive oil was purchased from Sigma Aldrich (Saint-Quentin-Fallavier, France). Composition of oils is summarized in table 1. Chemicals, cell culture reagents and fluorescent dyes were purchased from Sigma Aldrich, Eurobio (Les Ulis, France) and Invitrogen (Cergy Pontoise, France), respectively.

**Table 1:** Fatty acids (%) and tocopherol composition of tested oils

	Microalgae DHA	EPAX 1050	EPAX 6000	Refined Olive oil
C16:0 (palmitic acid)	20.6	3.03	6.04	12.10

C18:1 w9 (oleic acid)	14.0	6.52	4.82	70.31

C18:2 w6 (linoleic acid)	1.1	0.75	0.79	9.97

C20:5 w3 EPA	0.9	16.35	40.23	0.0

C22:6 w6 DHA	35.1	50.84	25.89	0.0

Mixed Tocopherol (mg/g)	2.0	3.8	3.8	0.0

### Animals

All procedures in this study complied with the Guide for the Care and Use of Laboratory Animals. Animal care and experimentation complied with the rules of European Council Guidelines: licence for experimental studies on living animals and agreement of animal facilities number A75-06-02 (Direction of Veterinary Services, Paris Police Department, France). 6-week old male Sprague-Dawley rats (Janvier, France) were used in the study. Animals were placed in cages (three rats per cage) and kept in standard laboratory conditions (20°C, 12 h/12 h light/dark cycle), fed *ad libitum *on the standard laboratory diet with a free access to water.

Fifteen rats were randomly and equally divided into five groups: control group, refined olive oil group, microalgae DHA oil group, EPAX 1050 oil group and EPAX 6000 group (Table 1). Oils were administered by gavage (1 mL/rat) every day for 4 weeks.

At the end of the protocol, euthanasia was produced using inhaled isoflurane and corneas were enucleated. Half-cornea sections were placed on 12-well plates and immerged in paraformaldehyde 4%.

### Cornea fatty acids composition

Corneas were enucleated and stored at -20°C in chloroform-methanol (2:1). The lipids were extracted according to Folch method [17]. Fatty acids were separated by gas chromatography as previously described [18].

### Cell Culture

A human corneal cell line (HCE, RCB 1384) was cultured under standard conditions in a mixture 1:1 of DMEM and Nutrient Mixture F12 supplemented with 10% foetal calf serum, 2 mM L-glutamine, 50 IU/ml penicillin and 50 IU/ml streptomycin. The medium was changed every 2 days. Confluent cultures were removed by trypsin incubation, and then the cells were counted. They were seeded into 96-well culture microplates at a density of 70,000 cells/well and kept at 37°C for 24 hours.

### Cell incubation

Whenever cells reached confluency, the culture medium was removed and the cells were exposed to the different oils for 15 minutes. After a 24-hour recovery time in culture medium [19], the cells were incubated with 50 μM tBHP (tert-butyl hydroperoxide) for 1 hour. Fatty acids were dissolved in ethanol and diluted in culture medium. The incubation time for fatty acids was 24 hours. The final ethanol concentration was 0.1% in culture medium, which doesn't affect cell viability (data not shown).

### Cytofluorometry Analysis

Experiments were conducted using microplate fluorometer (Safire, Tecanä, France), which allows fluorometric detection (280-870 nm) with high sensitivity (pg-fg/mL) and specificity. This technique allows the use of fluorescent probes directly on living cells and detects the fluorescent signal directly in the microplate in less than 1 minute (for a 96-well microplate).

#### Reactive oxygen species overproduction: DCFH-DA test

Reactive oxygen species were detected with the 2',7'-dichlorofluorescein diacetate probe. Once inside the cell, this probe is cleaved by endogenous esterases and can no longer pass out of the cell. The de-esterified product becomes the fluorescent compound 2',7'-dichlorofluorescein after oxidation by reactive oxygen species. Cells were incubated for 20 minutes with a 20 μM DCFH-DA solution, fluorescence detection was undertaken using λexc = 485 nm, λem = 535 nm.

#### Mitochondrial mass: NonylAcridine Orange test

Mitochondrial mass was evaluated using the NonylAcridine Orange probe (NAO), which stains lipids found exclusively in the mitochondrial inner membrane. Cells were incubated with the dye solution (10 μM in culture medium) for 30 minutes at 37°C. After a one hour recovery period, cells were washed in PBS and incubated with a solution of water (48.4%)-ethanol (50.6%)-acetic acid (1%). The plate was agitated on a microplate shaker for 30 minutes and the microplate was read at λexc = 490 nm, λem = 530 nm.

#### Mitochondrial potential: JC-1 test

Mitochondrial potential is measured using the JC-1 probe, which forms J-aggregates when mitochondrial activity is high (hyperpolarisation) and monomers when mitochondrial activity is low. The fluorescence emission of JC-1 depends on the probe state: aggregates produce a red fluorescence whereas monomers induce a green fluorescence. Cells were incubated in a dye solution at 10 μM for 15 minutes, fluorescence detection was undertaken using λexc = 485 nm, λem = 520 nm. Carbonyl cyanide *m*chlorophenylhydrazone (CCCP) was used as a positive control for mitochondrial depolarization.

### Data Analysis

Data in the text and graphs are shown as fluorescence percentage of the control, and statistical analysis were performed using a one-way ANOVA followed by a Dunnet's test (α risk = 0.05). Each test was performed in triplicate.

### Immunofluorescence for caveolin-1 expression

#### Confocal microscopy

Briefly, the culture cells were washed in PBS with 1% bovine serum albumin (BSA) for 10 minutes to block nonspecific binding. After permeabilization using Triton X100, the sections were incubated with primary antibodies in a solution of PBS-1% BSA and incubated for 1 hour at 4°C. The sections were incubated with the secondary antibodies diluted 1:500 (Alexa 488 goat anti-rabbit IgG). Nuclei were stained using propidium iodide.

#### Flow cytometry analysis

The culture cells were harvested with trypsin-EDTA, pelleted, washed twice in PBS, and fixed in paraformaldehyde. The cells were not permeabilized in order to study extracellular caveolin-1. The cells were incubated with anti-caveolin-1 antibody for 45 minutes. After wash, the cells were incubated with fluorescein isothiocyanate (FITC)-conjugated secondary antibody for 45 minutes. Flow cytometric quantitation of fluorescence was measured using Cytomics FC 500 flow cytometer (Beckman Coulter, France).

## Results

### *In vivo *effects of oils on corneal fatty acids composition

Rats were fed with microalgae DHA oil, fish EPAX 1050 oil, fish EPAX 6000 oil or refined olive oil. At the end of the protocol, the fatty acids composition of corneas was analysed using chromatography, results are presented in Table 2. Microalgae DHA oil and EPAX 1050 oil significantly decreased the ratio w6/w3 from 6.0 (control rats) to 2.5 (rats fed with microalgae DHA) and 3.6 (rats fed with EPAX 1050). Oleic acid was significantly increased from 21.2% (control rats) to 27.4% (rats fed with olive oil) after 28 days of gavage. Total MUFA was significantly increased from 30.9% (control rats) to 39.1% (rats fed with olive oil) and total PUFA was significantly decreased from 25.0% (control rats) to18.9% (rats fed with olive oil) after gavage.

**Table 2:** Fatty acids composition (%) of corneal membranes

	Control	Refined Olive oil	Microalgae DHA oil	EPAX 1050 oil	EPAX 6000 oil
**C18:1 w9** **(oleic acid)**	21.2 (0.22)	27.4 (0.68)***	23.1 (1.63)	23.0 (0.51)**	25.0 (0.46)*

**C20:4 w6 (arachidonic acid)**	13.8 (0.59)	9.4 (0.37)**	9.9 (2.05)	10.4 (0.58)*	10.5 (0.0)**

**MUFA**	30.9 (1.07)	39.1 (0.75)**	33.0 (2.59)	33.0 (0.93)	36.3 (0.22)*

**PUFA**	25 (0.78)	18.9 (0.4)**	22.0 (3.55)	21.3 (0.87)*	20.9 (0.44)*

**Ratio w6-w3**	6.0 (0.75)	7.3 (0.89)	2.5 (0.25)**	3.6 (0.35)**	4.7 (0.40)

MUFA: monounsaturated fatty acids, PUFA: polyunsaturated fatty acids

Rats were fed with oils during 28 days and after euthanasia, fatty acids of corneas were analysed using gas chromatography. * p < 0.01, **p < 0.005, ***p < 0.001 (according to Tukey's test).

### *In vitro *effects of oils and fatty acids on oxidative stress

**Figure 1:**
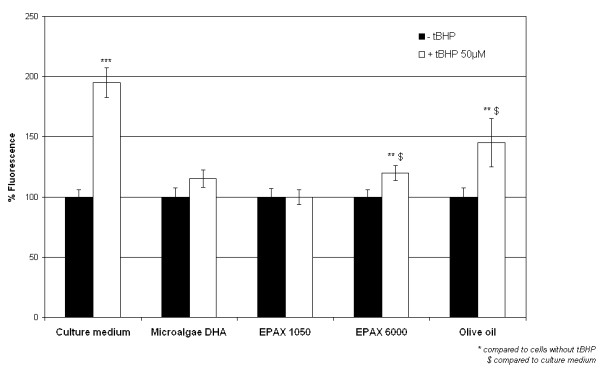
**Effects of oils on reactive oxygen species production by human corneal cells (H2DCF-DA test)**. The HCE-T cells were preincubated with oils or culture medium during 15 minutes followed by a 24-hour recovery time and then incubated with tBHP 50 μM or PBS during 1 hour. tBHP induced an overproduction of ROS partially or totally inhibited by the tested oils. *** *p *< 0.001, ** *p *< 0.01, $*p *< 0.001.

**Figure 2:**
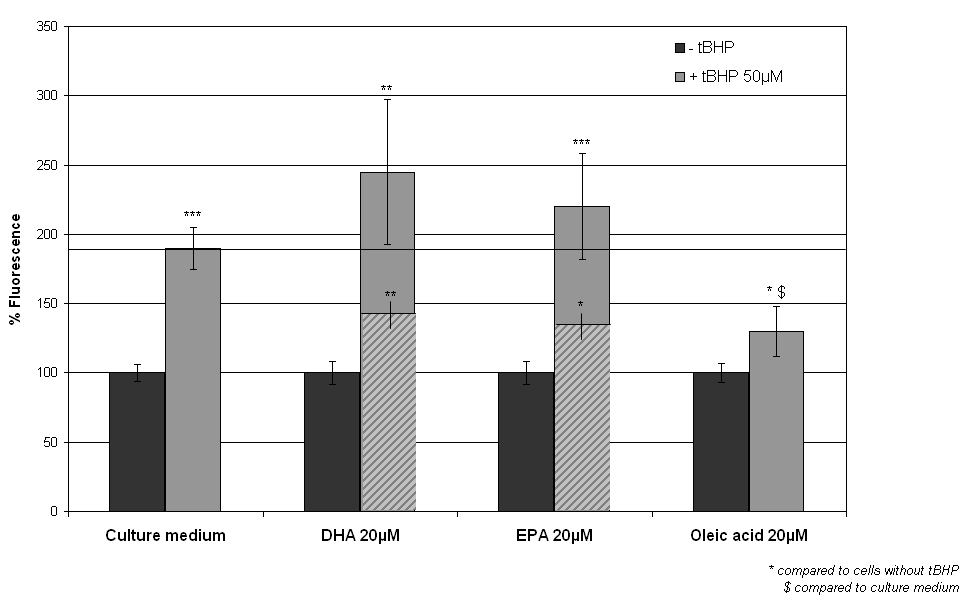
**Effects of fatty acids on reactive oxygen species production by human corneal cells (H2DCF-DA test)**. The HCE-T cells were preincubated with fatty acids during 24 hours and then incubated with tBHP 50 μM or PBS during 1 hour. Mixtures DHA+mixed tocopherols and EPA+mixed tocopherols were also tested for their ability to decrease oxidative stress (hatched columns). tBHP induced an overproduction of ROS partially inhibited by the mixtures and oleic acid. *** *p *< 0.001, ** *p *< 0.01, * $*p *< 0.001.

Since *per os *administered oils can reach the cornea, we investigated their effects on oxidative stress using an *in vitro *model of corneal cells. Our positive control, tBHP 50 μM-1 hour, induced a significant increase in reactive oxygen species production, almost 2× compared to culture medium control (figure 1). When the cells were preincubated with any of the tested oils, tBHP-induced reactive oxygen species generation was inhibited. Nevertheless, when the cells were preincubated with EPAX 6000 oil or olive oil, tBHP-induced reactive oxygen species production was partially inhibited (1.2× and 1.4× compared to control). The same range of inhibition was observed with oleic acid 20 μM (1.3× compared to control instead of 2× with culture medium, figure 2). On the contrary, preincubation of cells with DHA 20 μM or EPA 20 μM had no effect on tBHP-induced reactive oxygen species production (figure 2). When DHA and EPA were enriched in mixed tocopherols, tBHP-induced reactive oxygen species production was decreased from 245% to 140% for the mixture DHA-tocopherols and from 220% to 135% for the mixture EPA-tocopherols.

### *In vitro *effects of fatty acids on mitochondria

**Figure 3:**
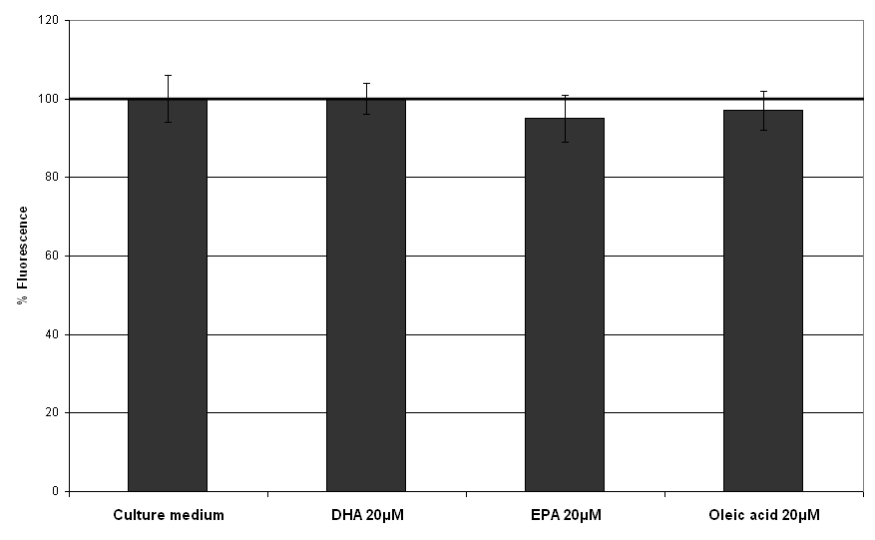
**Effects of fatty acids on mitochondrial mass of human corneal cells (NAO test)**. The HCE-T cells were incubated with fatty acids during 24 hours. None of the fatty acids we tested altered mitochondrial mass.

**Figure 4:**
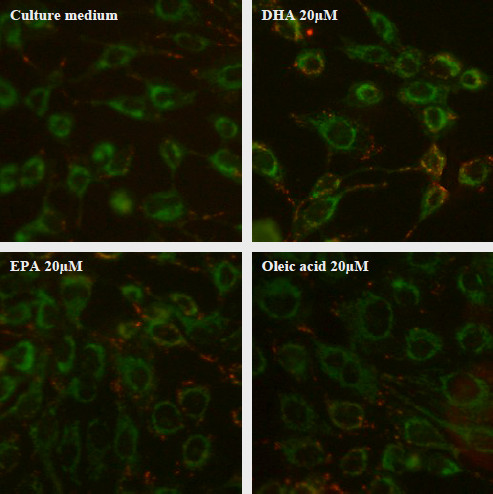
**Effects of fatty acids on mitochondrial transmembrane potential of human corneal cells (using JC-1 dye)**. The HCE-T cells were incubated with fatty acids during 24 hours. Green mitochondria are depolarized mitochondria and red mitochondria are hyperpolarized mitochondria. No change of mitochondrial transmembrane potential was observed.

To estimate the potential effects of fatty acids on mitochondria, mitochondrial mass and mitochondrial transmembrane potential were evaluated. DHA 20 μM, EPA 20 μM and oleic acid 20 μM had no effect on mitochondrial mass (figure 3). There was no significant difference between NAO fluorescence signals compared to control. Figure 4 shows JC1 fluorescence signal evaluating mitochondrial transmembrane potential. DHA 20 μM, EPA 20 μM and oleic acid 20 μM did not induce any variation of JC1 fluorescence compared to control. No matter which fatty acid was added (DHA, EPA or oleic acid), red mitochondria (hyperpolarized) were mainly distributed in the cytoplasm and green mitochondria (depolarized) were mainly distributed near the nucleus of cells.

### *In vitro *effects of fatty acids and oils on caveolin-1 expression

**Figure 5:**
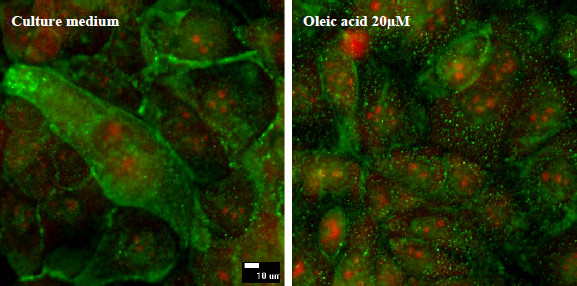
**Caveolin-1 immunoreactivity (confocal microscopy)**. The cells were incubated with oleic acid during 24 hours and fixed. After permeabilization, caveolin-1 was immunostained. The expression of caveolin-1 protein becomes more punctuate and less diffuse after oleic acid treatment, compared to control.

**Figure 6:**
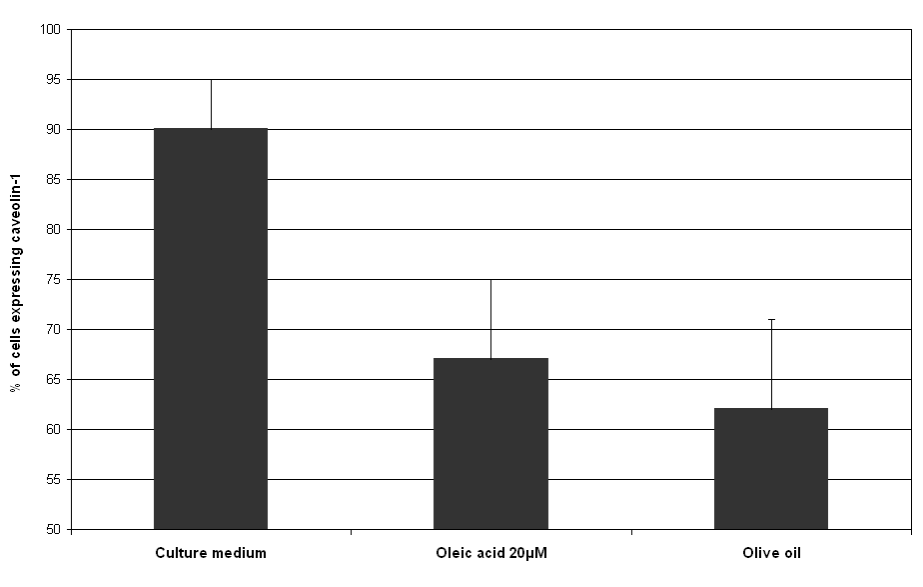
**Caveolin-1 immunoreactivity (flow cytometry)**. The cells were incubated with oleic acid during 24 hours or olive oil during 15 minutes followed by a 24-hour recovery time and fixed. Caveolin-1 was immunostained. Oleic acid and olive oil induce a loss of the protein.

To evaluate the effects of oleic acid on plasma membrane caveolae, caveolin-1 was immunostained and observed under confocal microscopy. Isotype control monoclonal antibody was used to estimate the non-specific binding of target primary antibody to cell surface antigens (data not shown). Since oleic acid showed antioxidant properties, its effects on caveolin-1 expression was studied. When the cells were incubated with oleic acid 20 μM, caveolin-1 expression was punctuated whereas control cells (culture medium) show a more diffuse staining (figure 5). Figure 6 shows caveolin-1 expression at the plasma membrane level using flow cytometry. Incubation of cells with oleic acid 20 μM for 24 hours or olive oil for 15 minutes followed by a 24-hour recovery time induced a decrease in caveolin-1 expression: 67% of cells incubated with oleic acid expressed caveolin-1 and 62% of cells incubated with olive oil expressed caveolin-1, 90% of control cells expressed caveolin-1.

## Discussion

To our knowledge, there is no published study on the incorporation of dietary fatty acids into the cornea. According to our study, fatty acids are able to reach the cornea after a *per os *supplementation. PUFA-rich oils decreased the ratio w6/w3 and refined olive oil increased the proportion of oleic acid in corneal cells of rats. At the cell level, consequences can be numerous; therefore, we investigated whether these oils exerted antioxidant properties *in vitro*. In our study, PUFA-rich oils (microalgae DHA, EPAX 1050 and EPAX 6000 oils) were able to decrease tBHP-induced oxidative stress. However, PUFA are highly susceptible to damaging, free radical initiated, lipid peroxidation particularly when intakes of antioxidants such as vitamin E, the major lipid peroxidation chain breaking antioxidant in membranes are low. Increased intakes of PUFA, without concomitant antioxidant supplementation have been shown to exceed the protective capacity of the antioxidant defence systems [20,21]. That is the main reason why the three of PUFA-rich oils we tested contain mixed tocopherols. Microalgae DHA and EPAX 1050 oils, which both showed the most potent antioxidant effect, are both rich in DHA fatty acid (35% and 51%, respectively); this component could be at the origin of the antioxidant effect we observed. Therefore, we tested the antioxidant effects of several fatty acids, including DHA. Our results showed that DHA 20 μM and EPA 20 μM did not decrease tBHP-induced reactive oxygen species overproduction. DHA and EPA enriched with tocopherols partially decreased oxidative stress but not as much as oils did. Appropriate proportions of DHA/EPA and tocopherols seem to be needed to observe the most potent antioxidant effects in our model. Besides, oily formulations probably increase the incorporation of PUFA and tocopherols into cells. Oleic acid, which is the major component of refined olive oil, exerted a significant antioxidant effect, like refined olive oil (no polyphenols). Several mechanisms can explain the antioxidant properties of a molecule. Some molecules, such as polyphenols, are able to scavenge radicals thanks to their aromatic cycles; these molecules give an electron to radicals that become stable with paired electrons. It is unlikely that oleic acid, which is a monounsaturated fatty acid (C18:1 w9), acts as a radical scavenger. Therefore, we hypothesized that oleic acid could play its antioxidant role through mitochondria. Mitochondria, where most of radicals are produced, are possible targets for antioxidant molecules. Oxygen normally serves as the ultimate electron acceptor of the respiratory chain and is reduced to water. However, electron leak to oxygen through complexes I and III can generate superoxide anion [22]. If mitochondria are the major source of intracellular reactive oxygen species and mitochondria are most vulnerable to oxidative damage, then it would be ideal to deliver the antioxidant therapy to mitochondria. The most common method for targeting compounds to mitochondria makes use of the potential gradient across the mitochondrial inner membrane [23]. Therefore, we performed two different tests to evaluate the effects of fatty acids on mitochondria. Our results showed that none of the fatty acids we tested (DHA, EPA and oleic acid) disturbed mitochondrial function. Indeed, there was no effect on neither mitochondrial mass nor mitochondrial transmembrane potential. Consequently, we focused our attention on cell membranes and most particularly on caveolae, where fatty acids can incorporate. According to our results obtained by confocal microscopy and flow cytometry, oleic acid modifies caveolin-1 expression in both qualitative and quantitative manners. We can conclude that oleic acid decreased caveolin-1 expression at the plasma membrane level and alter caveolin-1 expression within the cell. In response to oleic acid treatment, caveolin-1 can follow a pathway from the plasma membrane to lipid bodies [24]. The punctuated expression of caveolin-1 and the decreased expression at the plasma membrane level that we observed after oleic acid treatment can be due to a translocation into lipid bodies and confirmed Pol's results with a new method for caveolin-1 expression assessment (using flow cytometry). The complete reorganization of caveolin-1 within membrane could explain the antioxidant effect of oleic acid. A review by Parat and Fox suggested that reactive oxygen species are generated intracellularly to serve as second messengers [25]. Several of the receptors initiating a signal transduction cascade involving reactive oxygen species are concentrated in caveolae. The basal level of reactive oxygen species is dramatically reduced when rafts are disrupted by cholesterol extraction and it is restored in cholesterol-repleted cells [16]. In a model of fibroblasts, exposure to hydrogen peroxide induced overexpression of caveolin-1 and this up regulation was prevented by antioxidants such as lipophilic vitamin E [26]. Oleic acid seems to be a potent antioxidant agent, acting as a "caveolae disrupter". This property could be very useful in ophthalmology as cornea is often exposed to oxidative stress. According to our study, marine PUFA-rich oils and refined olive oil modulate oxidative stress. Oleic acid, which modulates caveolin-1 expression, could be added to oils rich in DHA/EPA in nutritional supplements to decrease oxidative stress on the ocular surface.

## Competing interests

The authors declare that they have no competing interests.

## Authors' contributions

HL carried out the confocal microscopy studies and participated in the design of the study. DR and AG carried out the gas chromatography studies and participated in the design of the study. JMW participated in the design of the study and participated in its coordination. PR participated in the design of the study. All authors read and approved the final manuscript.
